# Calibration and volunteer testing of a prototype contactless respiratory motion detection system based on laser tracking

**DOI:** 10.1002/acm2.14607

**Published:** 2024-12-20

**Authors:** Isnaini Nur Islami, Amar Ma'ruf Irfan Muhamadi, Wahyu Edy Wibowo, Aloysius Mario Yudi Putranto, Arief Sudarmaji, Fielda Djuita, Supriyanto Ardjo Pawiro

**Affiliations:** ^1^ Department of Physics Faculty of Mathematics and Natural Sciences Universitas Indonesia, Depok, West Java, 16424 Indonesia and Department of Radiation Oncology Dr. Cipto Mangunkusumo National General Hospital Central Jakarta Indonesia; ^2^ Department of Physics Faculty of Mathematics and Natural Sciences Universitas Indonesia Depok West Java Indonesia; ^3^ Department of Radiation Oncology Dr. Cipto Mangunkusumo National General Hospital Jakarta Indonesia; ^4^ Department of Radiation Oncology MRCCC Siloam Hospital Semanggi Jakarta Indonesia

**Keywords:** calibration, laser‐based respiratory motion detection system, volunteer test

## Abstract

**Purpose:**

The goal of this study was to assess the feasibility of a cost‐effective prototype of a laser‐based respiratory motion detection system utilizing a Leuze LDS for breath monitoring through calibration and volunteer tests.

**Methods:**

This study was performed using the Anzai AZ‐773 V and computerized imaging reference systems (CIRS) motion phantoms for calibration tests. The calibration of the laser‐based respiratory motion detection system involved spatial accuracy testing, amplitude calibration, and temporal accuracy. Volunteer testing was conducted on eight volunteers at the inferior end of the sternum and the abdomen area. The accuracy of the data recorded by the laser‐based respiratory motion detection system was validated against established clinical reference tracking systems namely real‐time position management (RPM) and Anzai AZ‐733 V system.

**Results:**

Calibration with an Anzai AZ‐773 V and CIRS phantoms demonstrated an average error of 1.17% ± 0.64% and an average amplitude calibration correlation coefficient of 0.975 ± 0.004. Volunteer tests, compared to the Anzai AZ‐733 V clinical system and RPM system, revealed average correlation coefficients for deep inspiration breath‐hold are 0.931 ± 0.02 and 0.936 ± 0.03, respectively, and for free breathing are 0.85 ± 0.07 and 0.77 ± 0.1, respectively.

**Conclusions:**

Overall, the data suggest that the in‐house laser‐based respiratory motion detection system performed well, with an error percentage below 10%. A reasonably good correlation coefficient was obtained, indicating that the readings obtained from the laser system are consistent with those set on the phantom and clinical respiratory motion detection systems. Although promising through the calibration process and volunteer tests, further studies are required to generate trigger data linked directly to computerized tomography and linear accelerator facilities, thereby advancing the clinical viability of this innovative laser‐based respiratory motion detection system.

## 1 INTRODUCTION

In 2020, World Health Organization (WHO) statistical data revealed a total of 19.3 million recorded cancer cases and 9.9 million reported fatalities. The most prevalent cancer is breast cancer, followed by lung and colorectal cancers. On the other hand, the leading cause of cancer death is lung cancer, followed by colorectal, liver, and stomach cancers.^1^ Notably, a substantial proportion of both prevalent cases and mortalities result from cancers in the chest and abdomen areas, which are influenced by respiratory dynamics. Intrafraction movements, encompassing motions from the respiratory system, skeletal muscles, heart, and gastrointestinal system, constitute significant factors in this context.^2^ Breathing‐induced movement introduces uncertainty in the chest and abdomen during radiation treatment, highlighting the challenge of managing intrafraction motion in these regions.^3^

In the treatment planning process, considering intrafraction movement during radiation treatment activities necessitates an additional margin for clinical target volume (CTV) expansion called the internal target volume (ITV), as recommended by the International Commission on Radiation Units and Measurements (ICRU) Report 62.^4^ This expansion aims to optimize the coverage of a moving target tissue at the expense of increasing the irradiation area in the volume of healthy tissue.^5^ Respiratory movement introduces artifacts in imaging modalities, causing errors in tissue imaging that can affect the accuracy of contouring the target and healthy organs, which directly affects the radiation dose.^2^ To mitigate these challenges, a respiratory motion detection system that strategically modulates beams during specific movement phases was introduced to reduce the dose to organs at risk (OAR) and enhance treatment precision.

Although commercially available external respiratory motion detection systems exist, many still rely on surrogate target systems such as real‐time position management (RPM) using an external box marker and the Anzai AZ‐733 V system employing a belt pressure sensor for chest or abdominal movement detection.^2^ However, placing a surrogate target, such as a box marker, in the RPM system can lead to an increased surface dose if positioned in a radiation area with low reproducibility of position from fraction to fraction.^6^ The increased surface dose can reach up to 45% and 235% by a novel‐marker respiratory motion surrogate and two‐marker RPM box, respectively, tested in reference 7, with the area of increased dose ranging from 9.7 to 71.7 cm^2^.^7^ Furthermore, some existing commercial respiratory motion detection systems require additional time for patient preparation, involve technical complexities and wiring, and may reduce patient comfort during intervention.^8^ All systems require time for patient preparation and training but the laser‐based respiratory motion detection system such as we describe may mitigate other clinical problems. A laser‐based respiratory motion detection system does not utilize surrogate targets that can obstruct the radiation field or increase the surface dose. In addition, this system operates without direct contact with the patients, thereby offering improved comfort. The Anzai laser‐based system has been commercially available and clinically used. Hoshina et al.^9^ using this system for visual guidance in performing Stereotactic Body Radiation Therapy (SBRT) for thoracoabdominal tumors under abdominal compression has shown excellent performance. However, there are limitations related to the cost. As a result, this study aims to develop a prototype laser‐based respiratory motion detection system that could serve as a more affordable alternative to the Anzai laser‐based respiratory motion detection system. The prototype is priced at approximately 2500 USD, which includes the stand holder but excludes the cost of connectivity to the linac and CT. By offering a more cost‐effective solution, we hope to enhance interest and awareness about respiratory motion detection or tracking systems in Indonesia.

In Indonesia, the adoption of respiratory motion detection systems in radiation treatment remains uncommon, owing to equipment costs, complexity, and connectivity problems. Therefore, in the preliminary phase, a simple prototype of a laser‐based respiratory motion detection system is developed, which has not yet reached the connectivity phase with CT and linac systems. This study aims to evaluate the feasibility of a cost‐effective prototype of a laser‐based respiratory motion detection system utilizing a Leuze LDS for breath monitoring through calibration and volunteer tests. This laser‐based respiratory motion detection system is expected to offer easier implementation at a more affordable cost without compromising accuracy and reproducibility.

Currently, several respiratory motion detection devices are available clinically which are applied to respiratory motion detection systems, including Catalyst (C‐RAD), AlignRT (Vision RT), and Identify (Varian). The equipment can utilize 1–3 cameras. Although a single camera is theoretically enough to reconstruct a known structured light pattern, using multiple cameras enhances the field of view (FOV), captures more features and surface gradients, and improves registration accuracy with the reference surface in 6 Degree of Freedom (DOF), allowing calculation of three translational and three rotational shifts.^10^ The Catalyst, AlignRT, and Identify surface imaging system is capable of respiratory motion detection system during both simulation (retrospective and prospective acquisition) and treatment and utilizes high‐definition cameras.^10^, ^11^ In the next research, we will feature a camera system to detect movement in both the superior‐inferior (SI) and left‐right directions. If our research demonstrates promising results, our next focus will be on integrating the prototype with CT and linac systems.

## 2 MATERIALS AND METHODS

### 2.1 Design systems

**FIGURE 1:**
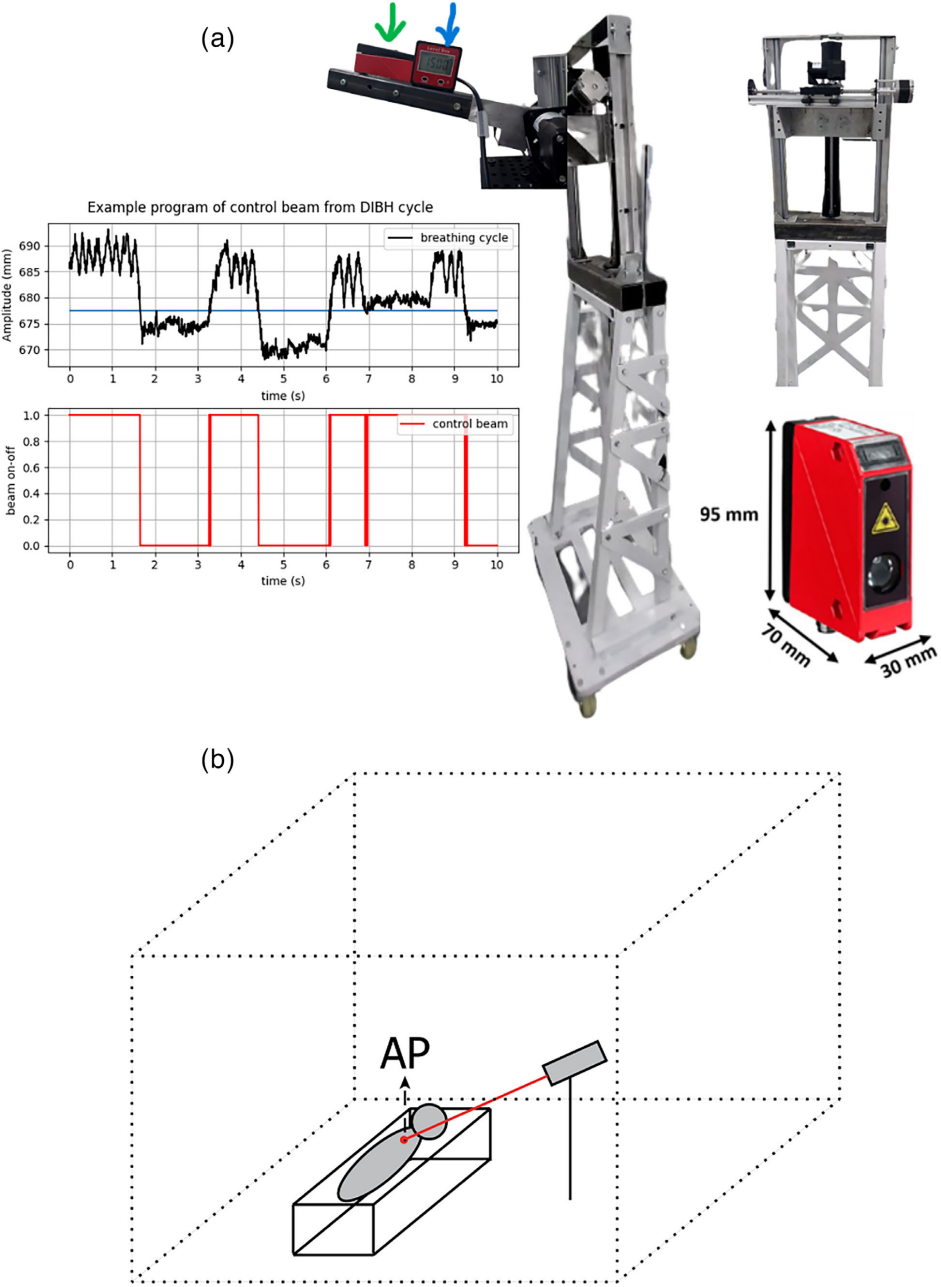
(a) Prototype in‐house laser‐based respiratory motion detection system, consisting of a Leuze LDS laser (red box with dimensions 70 × 30 × 95 mm^3^), an in‐house stand holder, and an interface created with a simple Python program. (b) Illustrate of recording and utilization of an in‐house laser‐based respiratory motion detection system. LDS, laser distance sensor.

The laser‐based respiratory motion detection system used in this study utilizes an LDS Optical Distance Sensor Laser (ODSL) 96 B (Leuze Electronic Corp., Germany). This laser distance sensor (LDS) measures the absolute distance between a fixed LDS and the patient's abdomen and sternum surface in real‐time during radiation treatment. The sternum and abdominal areas are chosen as appropriate substitutes for describing respiratory movements, with the sternum representing the thoracic region especially the superior end of the xiphoid or the inferior end of the sternum.^12^ and the abdomen encompassing the area between the xiphoid and umbilicus.^2^, ^13^ As shown in Figure 1a, the prototype incorporates in‐house stand holders strategically positioned to avoid obstructing the radiation field during the linac treatment. Importantly, this laser‐based respiratory motion detection system operates without physical interference with other systems. The LDS laser indicated by the green arrow in Figure 1a has a measurement range of 150–800 mm, spatial resolution of 0.1 mm, and response time of less than 15 ms. The in‐house stand holders, composed of metal, integrate a stepper motor linked to an Arduino Uno sandwiched with a Computer Numerical Control (CNC) shield equipped with an A4988 stepper motor driver. The device was connected to an Arduino Integrated Development Environment (IDE) application, providing time information for each distance read by the LDS. The LDS laser system was connected to a computer using a Universal Protocol Gateway (UPG) 10 cable, allowing the Python program to display real‐time data and enable amplitude by providing a threshold value to indicate the beam‐on and beam‐off statuses. However, this program only allows for displaying data and setting the beam on‐off threshold visually, as observed in Figure 1a, and has not yet reached the stage of implementing interacting with the treatment or imaging beam. The in‐house standholders represent a preliminary design aimed at testing the prototype performance of the laser‐based respiratory motion detection system affordably. Modifications to the in‐house standholder will be necessary for subsequent testing stages as visual guidance during CT imaging or treatment procedures on the linac. Further research is required to connect this in‐house LDS laser system to a linac or CT equipment.

### 2.2 Laser‐based respiratory motion detection system calibration

**FIGURE 2:**
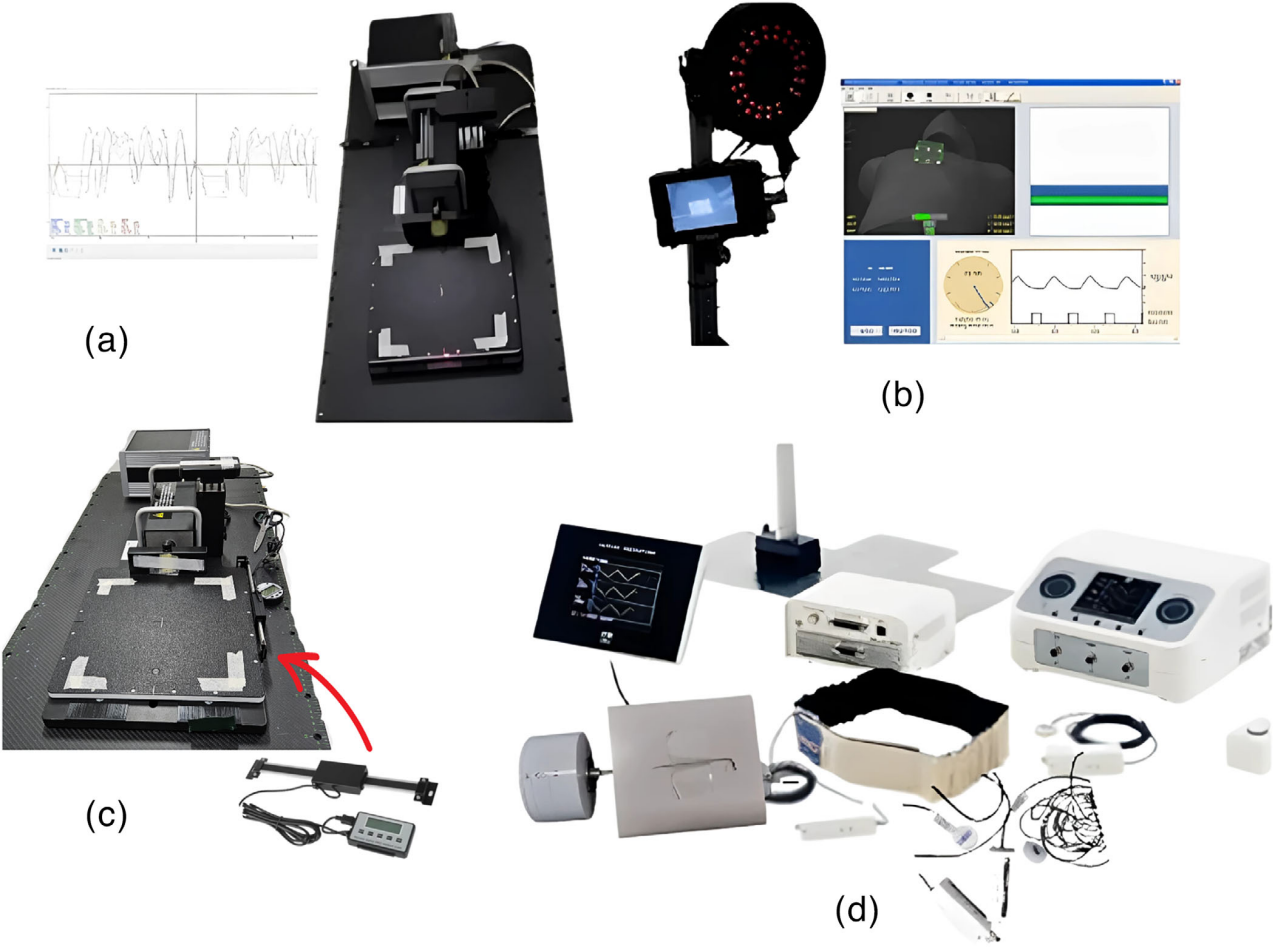
Equipment utilized in this study includes (a) Dynamic platform CIRS motion phantom set, which serves as a reference for amplitude calibration. (b) RPM, employed as a reference respiratory motion detection system to measure chest breathing movement simultaneously with the measure of breathing movement by the laser‐based respiratory motion detection system. (c) DLS, used as a reference for assessing spatial accuracy. (d) Anzai system set is used as a reference respiratory motion detection system for measuring abdominal breathing movement simultaneously with the measure of breathing movement by the laser‐based respiratory motion detection system. CIRS, computerized imaging reference systems; DLS, digital linear scale; RPM, real‐time position management.

The calibration of the laser‐based respiratory motion detection system in this study involved spatial accuracy testing, amplitude calibration with variations (5, 10, and 20 mm), and temporal accuracy using a dynamic platform model 008PL computerized imaging reference systems (CIRS) motion phantom (Sun Nuclear Corp., USA). The CIRS motion phantom, as shown in Figure 2a, which has an accuracy of 0.1 mm,^14^ has its cycle time or period adjusted based on the amplitude. In addition, amplitude calibration testing was conducted considering surface color variations using an Anzai motion phantom from Japan. The Motion Anzai phantom can be seen in Figure 2d, utilizes a standard load cell with high sensitivity to detect pressure via a strain gauge. When the sensor was attached to the belt, surface movements caused by breathing were visualized as breath waveforms. All tests were performed with a fixed LDS positioned perpendicular to the motion phantom. The measurement tests for calibrating this laser‐based respiratory motion detection system were varied to assess the accuracy.

A spatial accuracy test involves evaluating the percentage of measurement errors.^15^ Spatial accuracy measurements were performed using a CIRS motion phantom with a known amplitude. In the spatial accuracy test also, the readings of the phantom's movement from the initial point, or isocenter, to the peak or valley are evaluated as amplitude values. These values are then compared to the readings from the digital linear scale (DLS) as a reference to obtain deviation. This is similar to the static localization accuracy assessment for daily Quality Assurance (QA) tests as outlined in the American Association of Physicists in Medicine (AAPM) Task Group 302 (TG‐302) for surface‐guided radiation therapy.^10^ A DLS attached to a dynamic platform CIRS motion phantom was used to validate its movement as shown in Figure 2c. We tested 12 amplitude variations ranging from 2 to 24 mm, increasing by 2 mm increments. The selection of these amplitude variations is based on the average amplitudes during free‐breathing and deep‐inspiration breath hold of left‐sided breast cancer patients,^16^ expanded for comprehensive insights. A comparison was performed between the maximum point of the peak and valley, or twice the amplitude, between the known CIRS motion phantom values from the DLS readings and the values obtained from the LDS laser system readings. Subsequently, an analysis was performed using the percentage error equation as shown in Equation (1),^17^ with twice the amplitude of the DLS readings as the reference value and the LDS laser system readings as the experimental value.

(1) $$\begin{array}{cc} & \text{Percentage error}\\ & \;=\frac{X_{\text{measured by LDS laser}}-X_{\text{reference by DLS}}}{X_{\text{measured by LDS laser}}}\times 100 \end{array}$$

Amplitude calibration testing includes a review of the correlation coefficient and percentage of error from the root‐mean‐square error (PRMSE) calculations as shown in Equation (2). In this amplitude calibration, we varied the known and set amplitudes of the CIRS motion phantom, which were compared with the amplitude readings obtained using the LDS laser system. Additionally, the surface color of the phantom varied to analyze whether it affected the readings of the LDS laser. Several studies have revealed that the accuracy of laser system readings is influenced by surface color.^10^, ^18^

Temporal accuracy, a crucial aspect of respiratory motion detection systems, was determined by measuring the difference between the waveform period set by the phantom and the period read by the LDS. The temporal accuracy evaluation of the prototype laser‐based respiratory motion detection system in this study involved comparing the time taken to read one wave with the motion of the phantom motion circuit, employing a 10 s per wave setting. This approach was adapted from Jonsson et al.,^19^ where temporal accuracy was compared by assessing recorded frequencies against program‐frequency data in a phantom. In the AAPM Task Group 142,^20^ the expected temporal accuracy value was approximately 100 ms, assuming that the object movement occurred at a speed of no more than 20 mm/s.

The angle orientation starts from 0°, where the LDS views vertically down to the couch. This orientation is measured using an inclinometer, as shown blue arrow in Figure 1. This study varies 4 angles: 15°, 25°, 35°, and 45°. The angles were varied to determine the optimal angle for use during volunteer testing. This was performed to ensure that the laser position did not obstruct the radiation field area. This test was conducted in a manner similar to the amplitude calibration, with analysis using the percentage of error and correlation coefficient.

**FIGURE 3:**
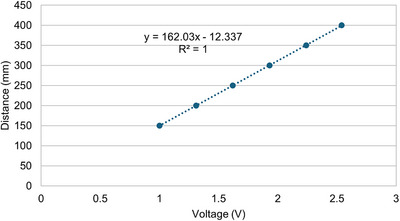
A linear equation as a conversion factor inputted into the Arduino Uno IDE program.

Manual calibration was performed before data acquisition to ensure that the laser readings were accurate and did not deviate. Manual calibration was performed to enhance the accuracy of laser readings. The user manual for the Leuze LDS system has an analog output ranging from 0 to 5 V, corresponding to an analog input ranging from 15 to 80 cm. We connected the Arduino board to the power supply of the LDS laser to read the analog laser measurements such that the analog output in the form of voltage could be displayed on the serial monitor of the Arduino Uno IDE as a distance. Consequently, we varied the distance readings with varying voltage outcomes and plotted the laser readings on the *y*‐axis against the voltage on the *x*‐axis, as shown in Figure 3. This enabled us to obtain a linear equation (y=mx+c), which was used as a conversion factor from the analog output to the real distance so that we could achieve accurate readings that correspond to the voltage outputs from the analog reading by the Leuze LDS system. We performed manual calibration when the readings on the laser LCD display differed from those displayed on the Arduino serial monitor. If they remain the same, we do not perform manual calibration, thus allowing the obtained linear equation to be used for the remaining studies, so it does not need to be done frequently.

### 2.3 Volunteer testing of the laser‐based respiratory motion detection system

**FIGURE 4:**
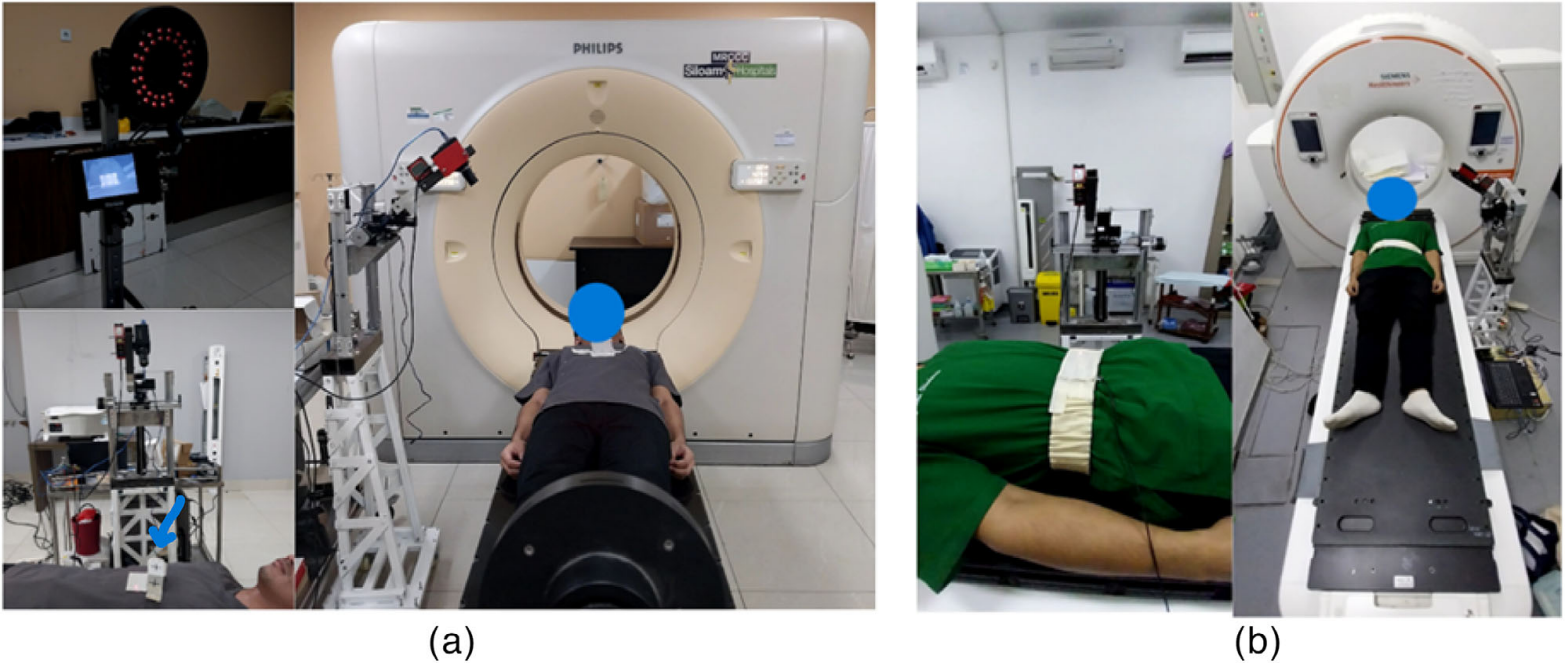
Volunteer setup with (a) RPM system at the inferior end of the sternum of the chest measurement area, (b) Anzai system at the abdomen measurement area. RPM, real‐time position management.

This study included healthy human subjects and was approved by the Ethics Committee. The test involved eight healthy volunteers, four males and four females, who were selected based on parameters such as body mass index (BMI) and waist circumference are listed in Table 1. Respiratory recordings using the laser system were conducted at two locations: the inferior end of the sternum area and the abdomen between the xiphoid and umbilicus, following the research by Kim et al.^12^ and Giraud et al.^13^ Respiratory recordings using the laser system were obtained at 35° angles at both locations to ensure an angle that would not obstruct the radiation field in a clinical situation. The accuracy of the data recorded by the laser‐based respiratory motion detection system was validated against established clinical reference respiratory motion detection systems. The reference systems used include RPM (Varian) for measuring the area under or inferior end of the sternum and Anzai (Anzai Medical) for measuring the abdominal area (area between the xiphoid process and the umbilicus), as shown in Figure 4. RPM system monitors the movement of the infrared‐reflecting marker block placed on the patient's surface, captured by a camera, which can be seen in Figure 4a. The placement of the block marker must be in an area that provides clear respiratory traces, and its positioning should be consistent between simulation and treatment. The application of the Anzai system using an elastic belt containing pressure sensors on the abdomen of the volunteer, can be seen in Figure 4b. The use of tight shirts was for the volunteers' modesty and comfort during the measurement can be seen in Figure 4, but for LDS measurements, it is better to be done directly on the actual patient surface.

**TABLE 1:** Subject characteristics (data are expressed as mean ± standard deviation (range).

A summary of volunteers (*n* = 8)		
	Female	Male
Age (year)	30.25 ± 2.86 (28–35)	28 ± 1.41 (26–30)
Waist circumference (cm)	74.75 ± 4.44 (69–80)	80 ± 5.43 (72–87)
BMI	21.52 ± 0.82 (20.1–22.1)	21.92 ± 1.66 (19.2–22)

Abbreviation: BMI, body mass index.

Verification is done after data collection, involving marking with a marker on micropore tape at the previous placement sites (inferior end of the sternum area during RPM system use and abdomen area with the Anzai system as the belt position) and performed also specifically during the use of the RPM system by noting the longitudinal and lateral positions of room lasers adjusted to align with the cross‐hair on the box marker of RPM system. The laser reflection point is placed close to the RPM marker, following the marker for the previous block position, and aligned with the center of the belt marked during simultaneous measurements with the Anzai system. Additional data is needed after verification by examining the breathing patterns from volunteers data. If deviations are observed, such as coughing during the breathing pattern or mismatched timing between the laser data and the clinical respiratory motion detection system data, the measurements are repeated. Two volunteers required remeasurement due to coughing and movement during the measurement, which caused the recorded breathing patterns to be irregular and inconsistent. Although the measurements are taken on the same day, there is inherent uncertainty regarding the positioning of the volunteers. The LDS and commercial system measurements are done simultaneously to assess the accuracy of the reading. Therefore, further research requires an assessment of the reproducibility and reliability of laser monitoring with data collection on the same subject at different times with measurement simultaneously with commercial respiratory motion detection systems. The validation process aimed to ensure that the prototype of this simple laser‐based respiratory motion detection system demonstrates satisfactory performance in recording respiratory movements, laying the foundation for further testing to develop a contactless respiratory motion detection system that is both easy to implement and effective. The analysis of the laser‐based respiratory motion detection system in this volunteer test included assessing the error with RMSE calculations as shown in Equation (2)^17^ and the correlation coefficient in Equation (3) between the laser‐based respiratory motion detection system prototype and the clinical respiratory motion detection system as well as determining the average shifting time results as a time delay from four volunteers. Time‐delay analysis utilized a Python application with the fast Fourier transform function code from Redaelli,^21^ transforming the time domain into the frequency domain and subsequently inverting it back to the time domain to obtain the time delay.

(2) $$\begin{array}{cc} \mathrm{RMSE} & =\sqrt{\frac{1}{n}\sum_{i=1}^{n}{\left(X_{i}-Y_{i}\right)}^{2}}\\ \mathrm{PRMSE} & =\sqrt{\frac{1}{n}\sum_{i=1}^{n}{\left(X_{i}-Y_{i}\right)}^{2}}\times 100 \end{array}$$

(3) $$r=\frac{n\sum_{i=1\;}^{n}X_{i}Y_{i}-\left(\sum_{i=1}^{n}X_{i}\right)\left(\sum_{i=1}^{n}Y_{i}\right)}{\sqrt{\left[\left(n-1\right)\sum_{i=1}^{n}X_{i}^{2}\right]\;\left[\left(n-1\right)\sum_{i=1}^{n}Y_{i}^{2}\right]}\;}\;$$

The best RMSE value is zero.^22^ Here, *n* represents the number of points for variables *X* and *Y*.^23^ We used the fast Fourier transform (FFT) function in Python. The equation used is as follows:

(4) $$Y\left(f\right)=\int_{-\infty }^{\infty }y\left(t\right)e^{i2\pi f}dt$$

where Y(f) represents the modified frequency domain transformation. Y(t) represents the breathing motion reading from the laser‐based LDS or clinical respiratory motion detection system in the time domain. Upon transforming the frequency domain, the next step is to convert it back to the time domain. This process involves taking the real part of the inverse transformation because only the real part is relevant for representing the signal in the time domain. The formula for converting back to the time domain is inverse fast Fourier transform.

(5) Y(t)=real{IFFT[Y]}

where Y(t) represents the signal in the transformed time domain. Y(f) represents the result of the modified frequency‐domain transformation. Subsequently, the initial time shift is initialized with the reading time from the respiratory motion detection system when reading each data point, and a loop for error calculation was conducted. The error between the shifted laser readings and the reference data was computed. This error represents the difference between the two datasets (laser system data readings $Y_{laser}\left[i\right]$ and clinical respiratory motion detection system data readings $Y_{reference}\left[i\right]$, all of which are signals in the time domain). We found the index of the minimum error as the optimal time shift that minimizes the error between the laser readings and reference data.

(6) $$\mathrm{Error}=\sum_{i=1}^{n}{\left(Y_{laser}\left[i\right]-Y_{reference}\left[i\right]\right)}^{2}$$

## 3 RESULTS

**FIGURE 5:**
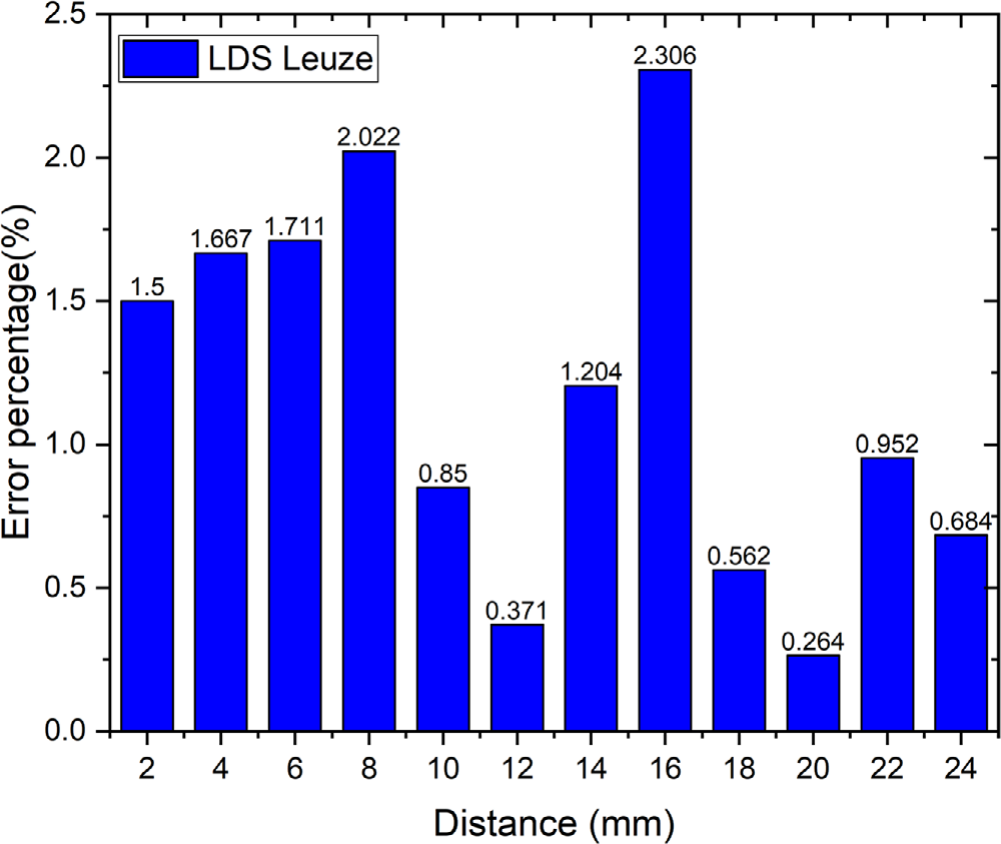
Spatial accuracy of Leuze LDS with amplitude variations. LDS, laser distance sensor.

Figure 5 shows the spatial accuracy of the Leuze LDS with 12 different constant amplitudes, namely 2, 4, 6, 8, 10, 12, 14, 16, 18, 20, 22, and 24 mm, at a distance of 35 cm from the Leuze LDS to the surface of the CIRS phantom. The error percentage from the 12 variations obtained the highest error of more than 1.5% at an amplitude of 16 mm, followed by 8, 6, and 4 mm, corresponding to 2.31%, 2.02%, 1.71%, and 1.67%, respectively, with an overall average error percentage value of 1.17% ± 0.64%. This study assessed the reproducibility of the laser system in calculating the coefficient of variation, yielding an average of 0.3%.

**FIGURE 6:**
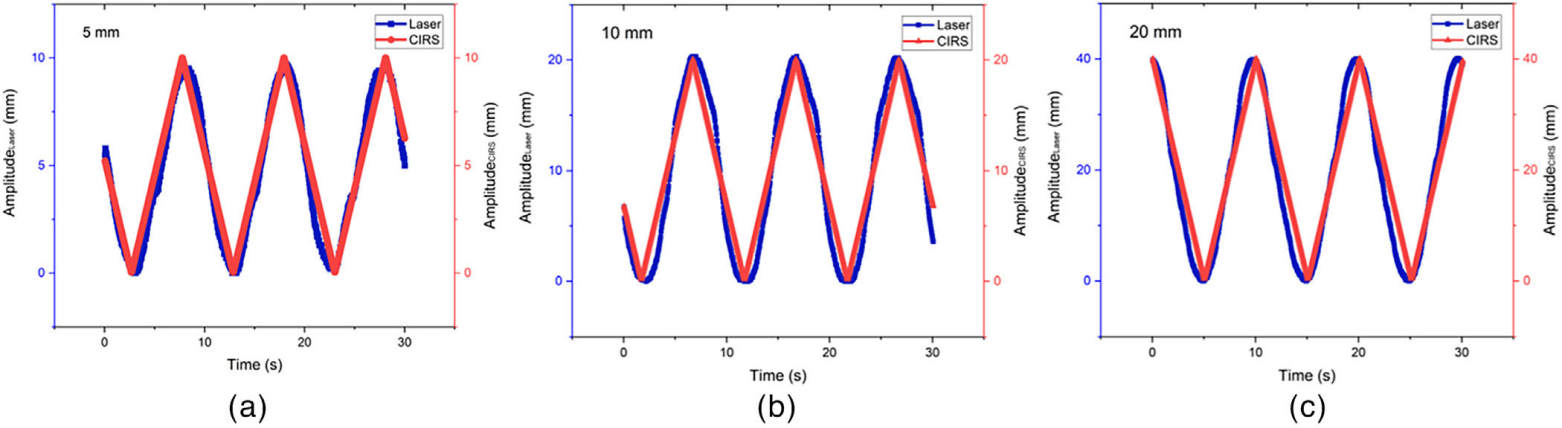
Result of laser and CIRS phantom readings with amplitude (a) 5 mm, (b) 10 mm, and (c) 20 mm. CIRS, computerized imaging reference systems.

Figure 6 shows the variations in amplitude calibration, namely 5 mm (a), 10 mm (b), and 20 mm (c), which were performed on the Leuze LDS against the CIRS phantom as a reference in approximately 30 s. The blue line represents the waveform of the laser‐based LDS system and the red line represents the waveform of the CIRS motion phantom. The quantitative results of the amplitude calibration presented in Table 2 indicate that the correlation coefficients are highest for 5 mm, followed by 10 and 20 mm. Conversely, the error percentage results are highest for 20 mm, followed by 10 and 5 mm. The average correlation coefficient between the laser system and the CIRS phantom is 0.975 ± 0.004, with an average error percentage of 0.22% ± 0.13%.

**TABLE 2:** Error percentage and correlation coefficient of the amplitude calibration result.

Amplitude CIRS	5 mm	10 mm	20 mm
Error percentage (%)	0.071	0.20	0.39
Correlation coefficient	0.981	0.976	0.97

Abbreviation: CIRS, computerized imaging reference systems.

**FIGURE 7:**
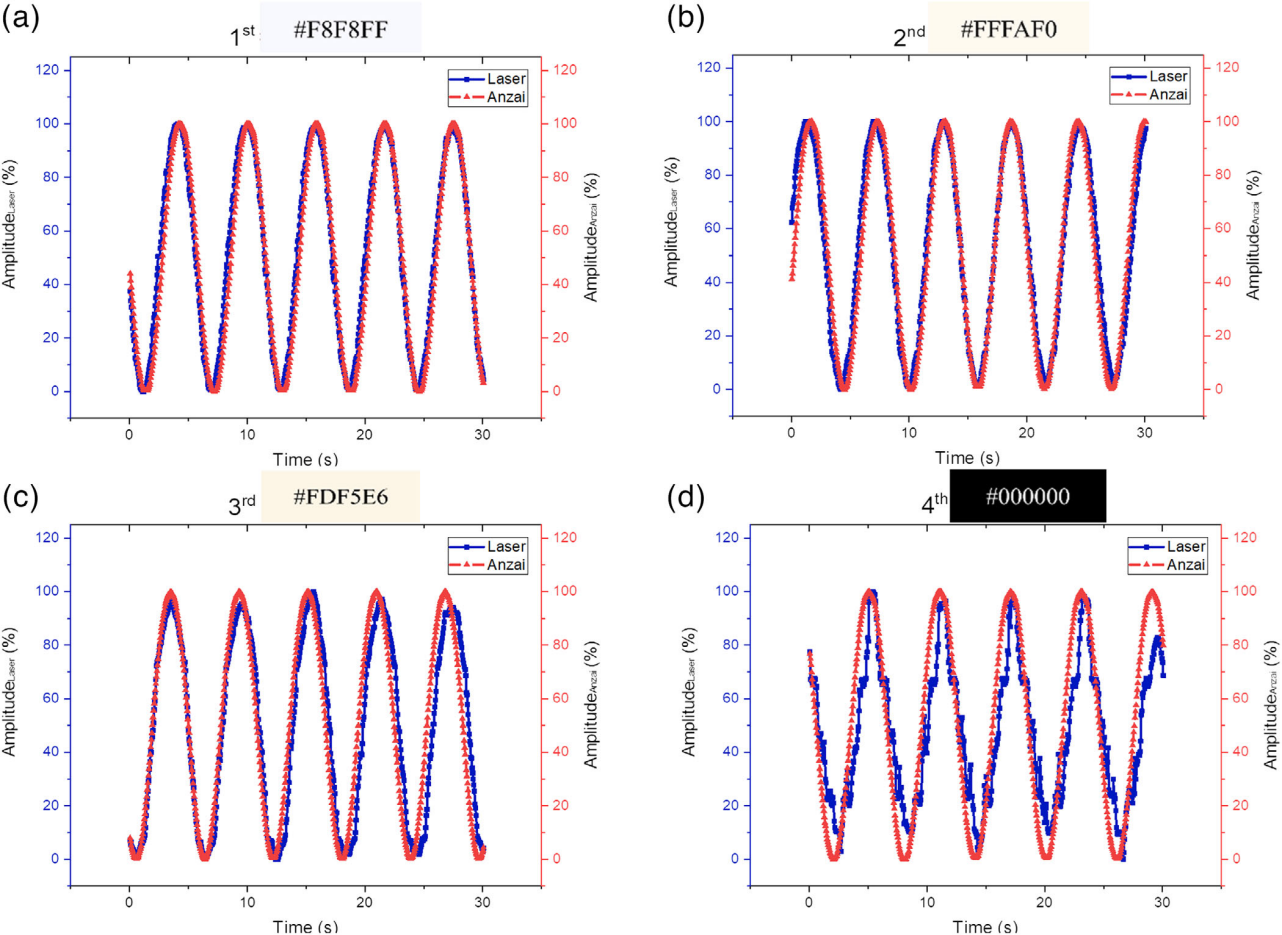
Measurement results of laser readings against the movement of the Anzai phantom on a variation of color surface.

Figure 7 and Table 3 demonstrate the impact of variations in the phantom surface color on the tracking readings using LDS. Figure 7a represents the waveform resulting from laser reading of the Anzai phantom motion with a white color (#F8F8FF) surface characterized by a soft surface, having a correlation coefficient of 0.983 with a percentage of error of 0.136%. Figure 7b illustrates the waveform from the reading laser to a floral white color (#FFFAF0) surface of the Anzai phantom, which has thicker characteristics than 8A with a correlation coefficient of 0.975 with a percentage of error of 0.157%.

**TABLE 3:** Correlation coefficient and error of Leuze LDS against Anzai phantom on a variation of color surface.

Color code	Percentage of error (%)	Correlation coefficient
#F8F8FF	0.136	0.983
#FFFAF0	0.157	0.975
#FDF5FF	0.253	0.936
#000000	0.336	0.901

Abbreviation: LDS, laser distance sensor.

Figure 7c depicts a color like 8B with the same characteristics but a slightly darker color (#FDF5E6), with a correlation coefficient of 0.936 and a percentage of error of 0.253%. Figure 7d shows the waveform results from reading a black‐colored (#000000) surface of the Anzai phantom with a slightly textured surface, with a coefficient correlation of 0.901 and a percentage error of 0.336%. Table 3 indicates that the #F8F8FF color has the smallest percentage of error and the highest correlation coefficient compared to the other colors, followed by the #FFFAF0, #FDF5E6, and #000000 colors.

The temporal accuracy of the prototype laser‐based system in this study obtained an average time deviation of 0.056 ± 0.02 s, resulting in an error percentage of 0.86% at a speed of 2 mm/s.

**FIGURE 8:**
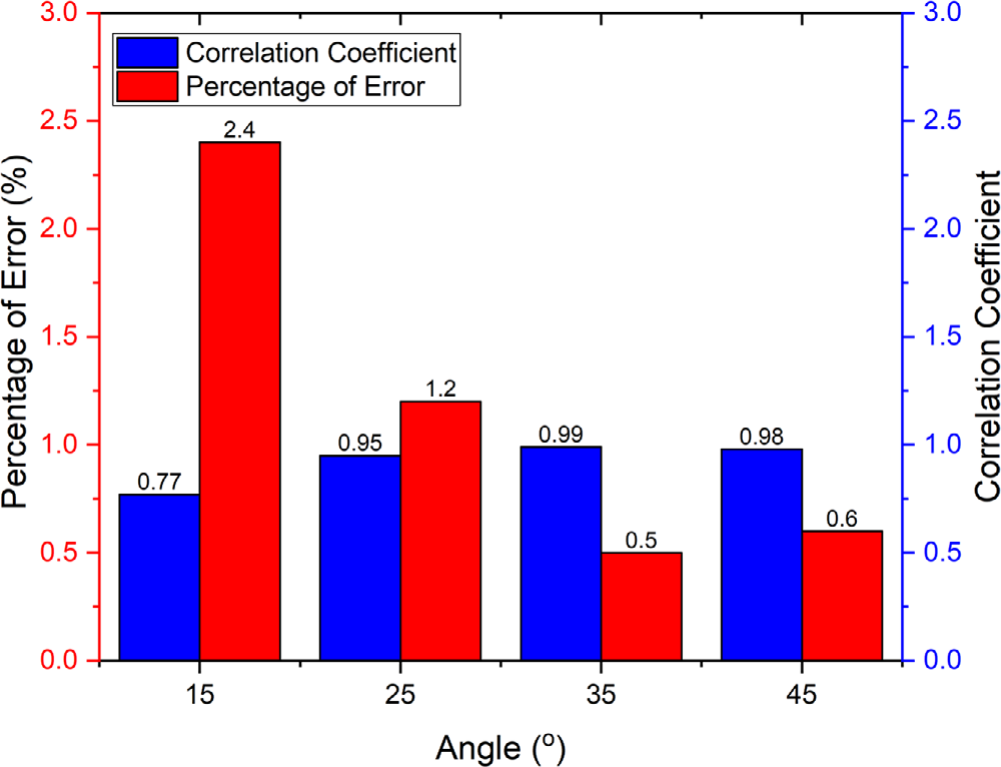
Correlation coefficient and percentage of error on variation in degree position of LDS. LDS, laser distance sensor.

Figure 8 illustrates that the correlation coefficient approaches one as the LDS angles toward the CIRS phantom increase and the error percentage decreases. Therefore, this study uses a 35° angle to collect laser data from volunteers in the volunteer tests because it resulted in a smaller error percentage and a greater correlation coefficient.

Figure 9 shows the breath waveforms of eight volunteers obtained from the Leuze LDS readings compared to the Anzai system readings. This breath waveform was recorded on the volunteer's abdomen. Volunteers with code F are female, whereas those with code M are male. The red line represents the laser reading, and the blue line represents the Anzai system reading. As shown in Figure 9a Deep Inspiration Breath Hold (DIBH), the amplitudes of the Anzai and laser were almost the same for female volunteers compared to male volunteers, where the Anzai readings were almost always lower than the laser readings for male volunteers. (Figure 9b) Free‐breathing shows that the breathing pattern read by the laser is similar to that of the Anzai system, but the amplitude is slightly different for all volunteers, both female and male.

FIGURE 9Comparison of the Leuze LDS and Anzai system measurements regarding DIBH and FB for each volunteer. Top (a): approximately 115–120 s with repetition of DIBH three times. Bottom (b): FB for approximately 30 s. LDS, laser distance sensor.

**Figure:**
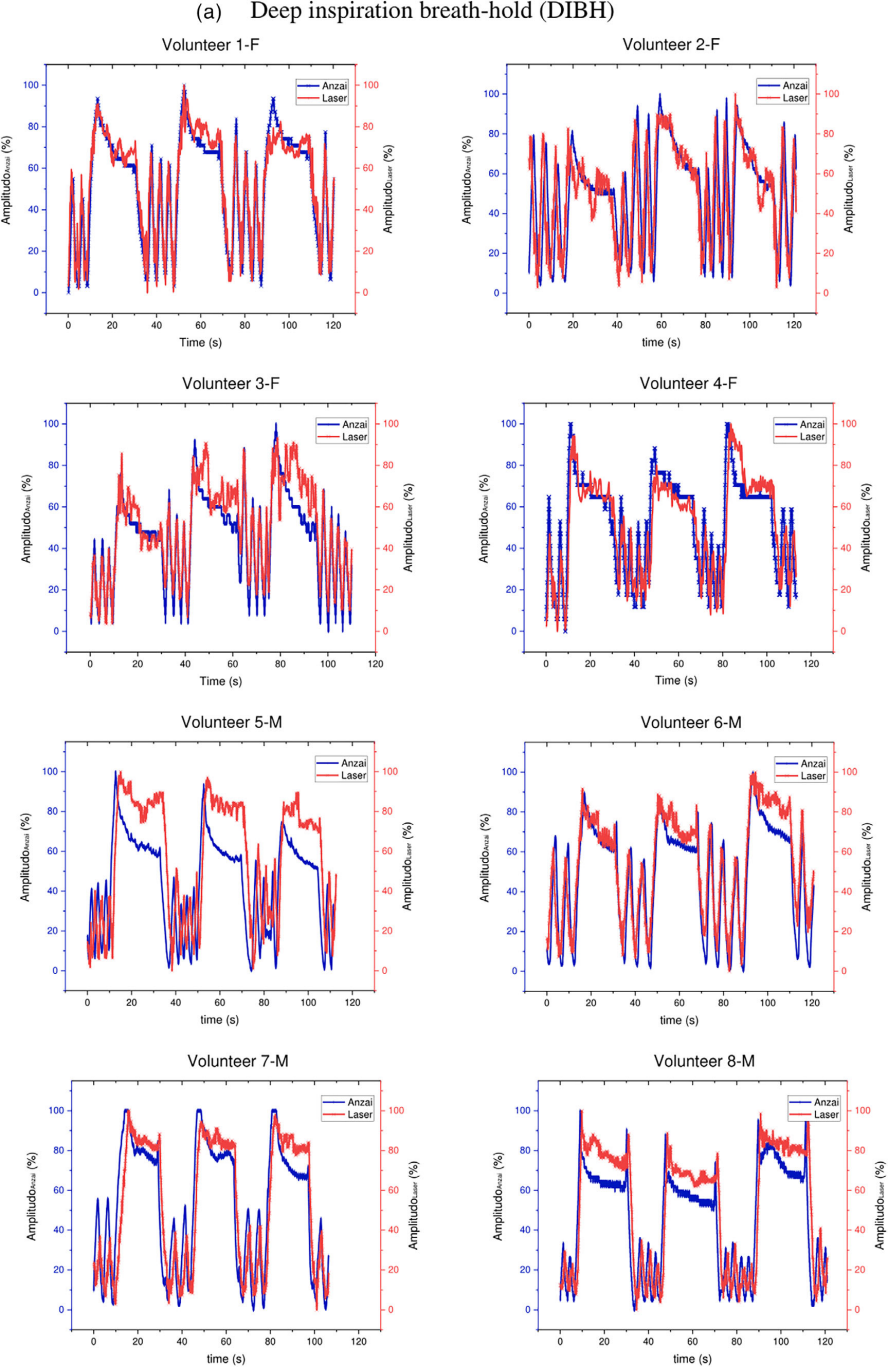

**Figure:**
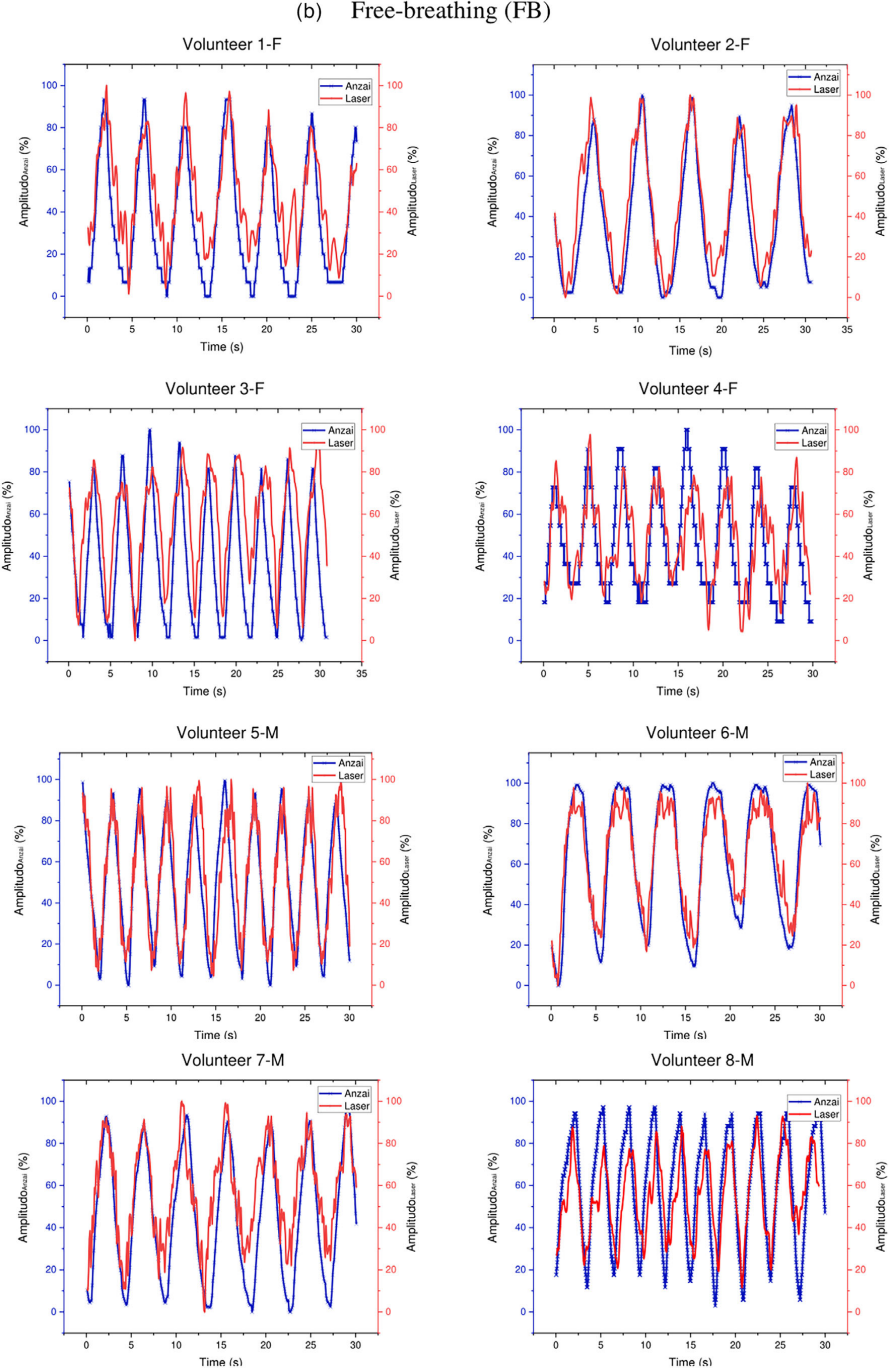

**FIGURE 10:**
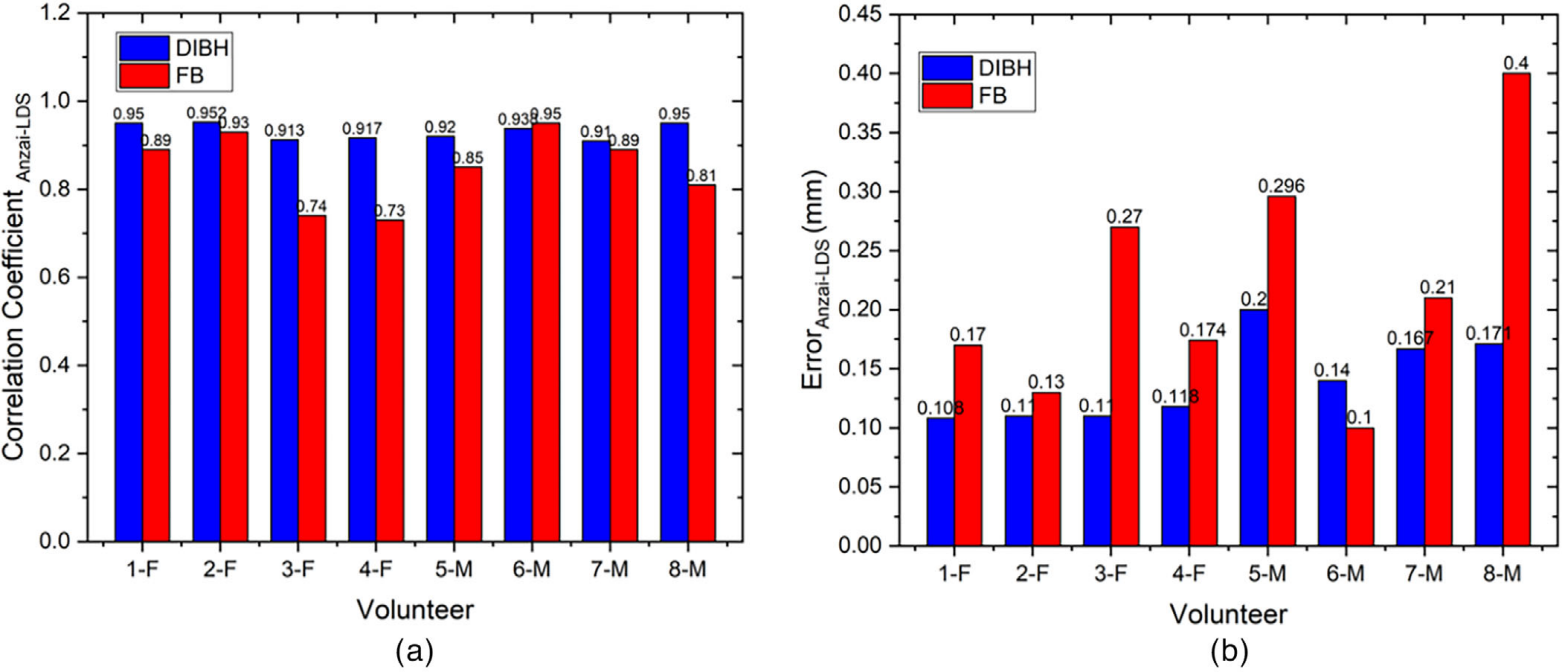
(a) Correlation coefficient and (b) error (RMSE) of Anzai system and LDS system for DIBH and FB of volunteers. LDS, laser distance sensor; RMSE, root‐mean‐square error.

Figure 10 shows the correlation coefficients and error values between the LDS and Anzai systems for the Free Breathing (FB) and DIBH techniques for each volunteer. Figure 10a explains that the correlation coefficient for DIBH is higher than that for free‐breathing for all volunteers, except for the 6‐M volunteers. The average correlation coefficient is 0.931 ± 0.02 for DIBH and 0.851 ± 0.07 for FB when compared with the LDS with the Anzai system Figure 9b explains that the error for free‐breathing is higher than that for DIBH for all volunteers, except for the 6‐M volunteer. The comparison between LDS and Anzai yielded an average error of 0.218 ± 0.09 mm and 0.14 ± 0.03 mm for FB and DIBH, respectively. Volunteer 6‐M has a slightly higher free‐breathing correlation coefficient and a smaller free‐breathing error percentage than DIBH.

**FIGURE 11:**
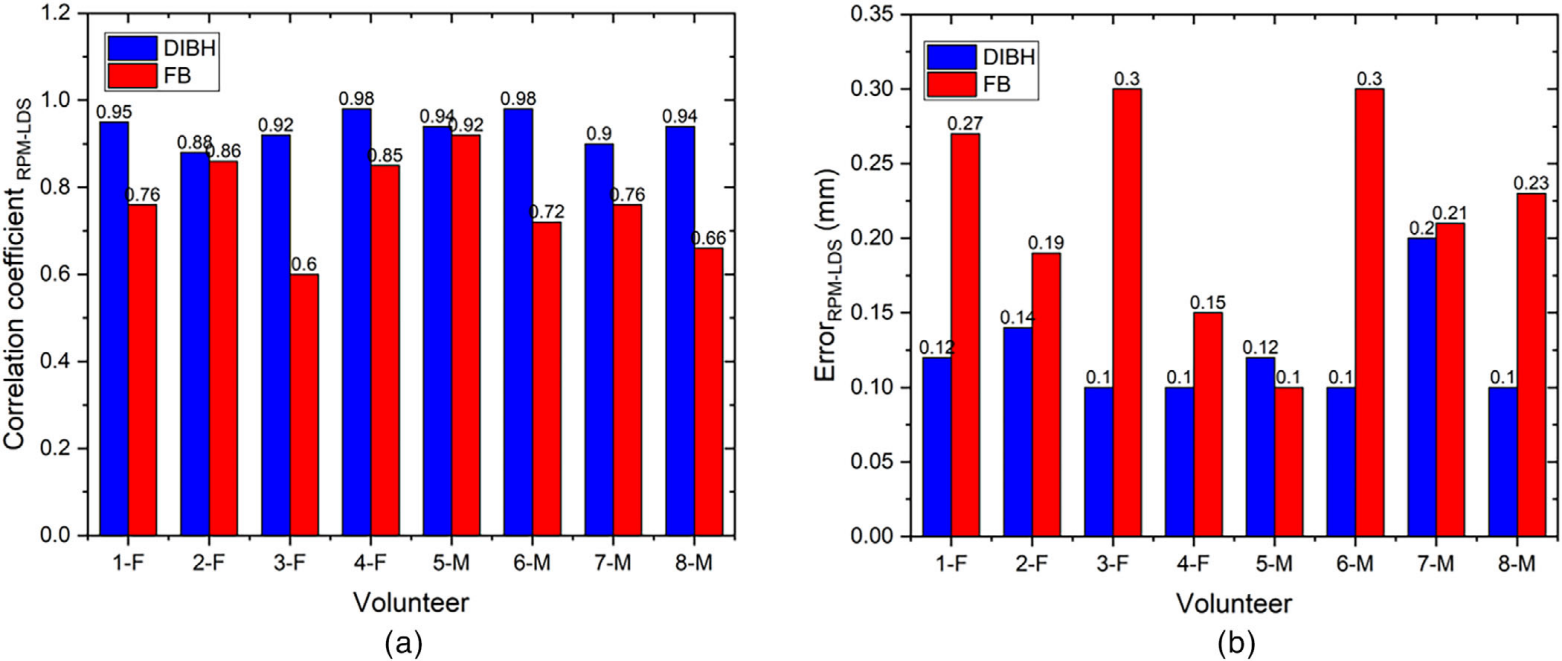
(a) Correlation coefficient and (b) error (RMSE) of RPM system and LDS system for DIBH and FB of volunteers. LDS, laser distance sensor; RMSE, root‐mean‐square error; RPM, real‐time position management.

Figure 11 shows the correlation coefficients and error values between the LDS and RPM systems for each volunteer using the FB and DIBH techniques. Figure 11a describes that the correlation coefficient for DIBH is higher than that for free‐breathing, whereas Figure 11b shows that the error for free‐breathing is higher than that for DIBH, for all volunteers. The average correlation coefficient is 0.936 ± 0.03 for DIBH, whereas 0.77 ± 0.1 for FB when comparing the LDS with the RPM system. In contrast, the comparison between LDS and RPM resulted in an average error of 0.22 ± 0.07 mm for FB and 0.12 ± 0.03 mm for DIBH. The DIBH error is slightly higher for 5‐M.

Figure 12 illustrates the breath waveforms of the eight volunteers obtained from the Leuze LDS readings compared with the RPM system readings. The breath waveform was captured from the volunteer's inferior end of the sternum, which is located in the thoracic region. The red line represents the laser reading and the blue line indicates the reading of the RPM system. As shown in Figure 12a DIBH the breathing patterns of all volunteers recorded by the laser are the same as those of the RPM system, but the RPM amplitude is different from that of the laser for all volunteers. The RPM amplitude is much greater than that of the laser in both the male and female volunteers. (Figure 12b) Free‐breathing also shows that the breathing pattern read by the laser is similar to that of the RPM system, but the amplitude is slightly different for all volunteers.

FIGURE 12Comparison of the Leuze LDS and RPM system measurements for DIBH and FB of each volunteer. Top (a): approximately 115–120 s with repetition of DIBH three times. Bottom (b): FB for approximately 30 s. LDS, laser distance sensor; RPM, real‐time position management.

**Figure:**
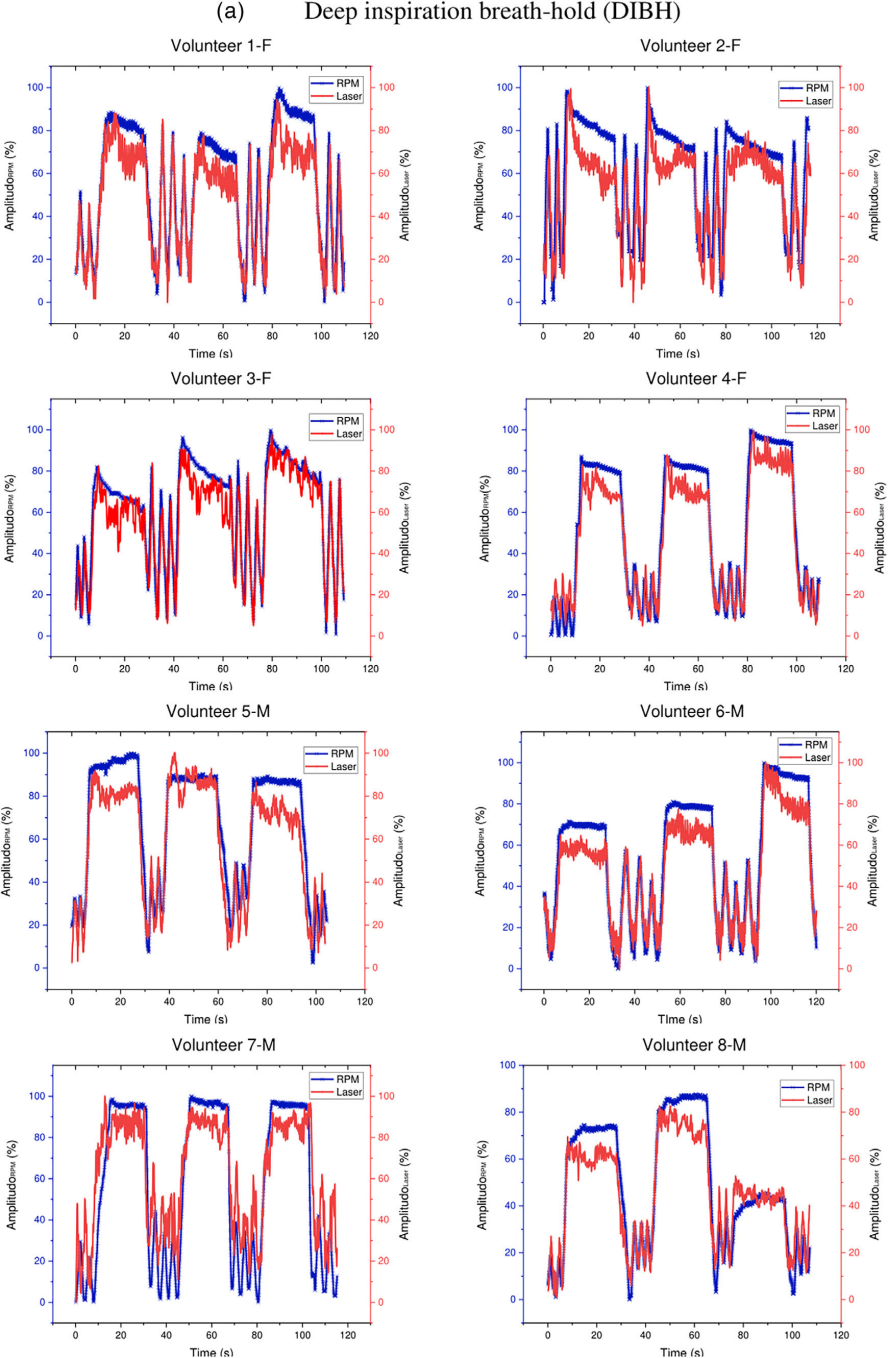

**Figure:**
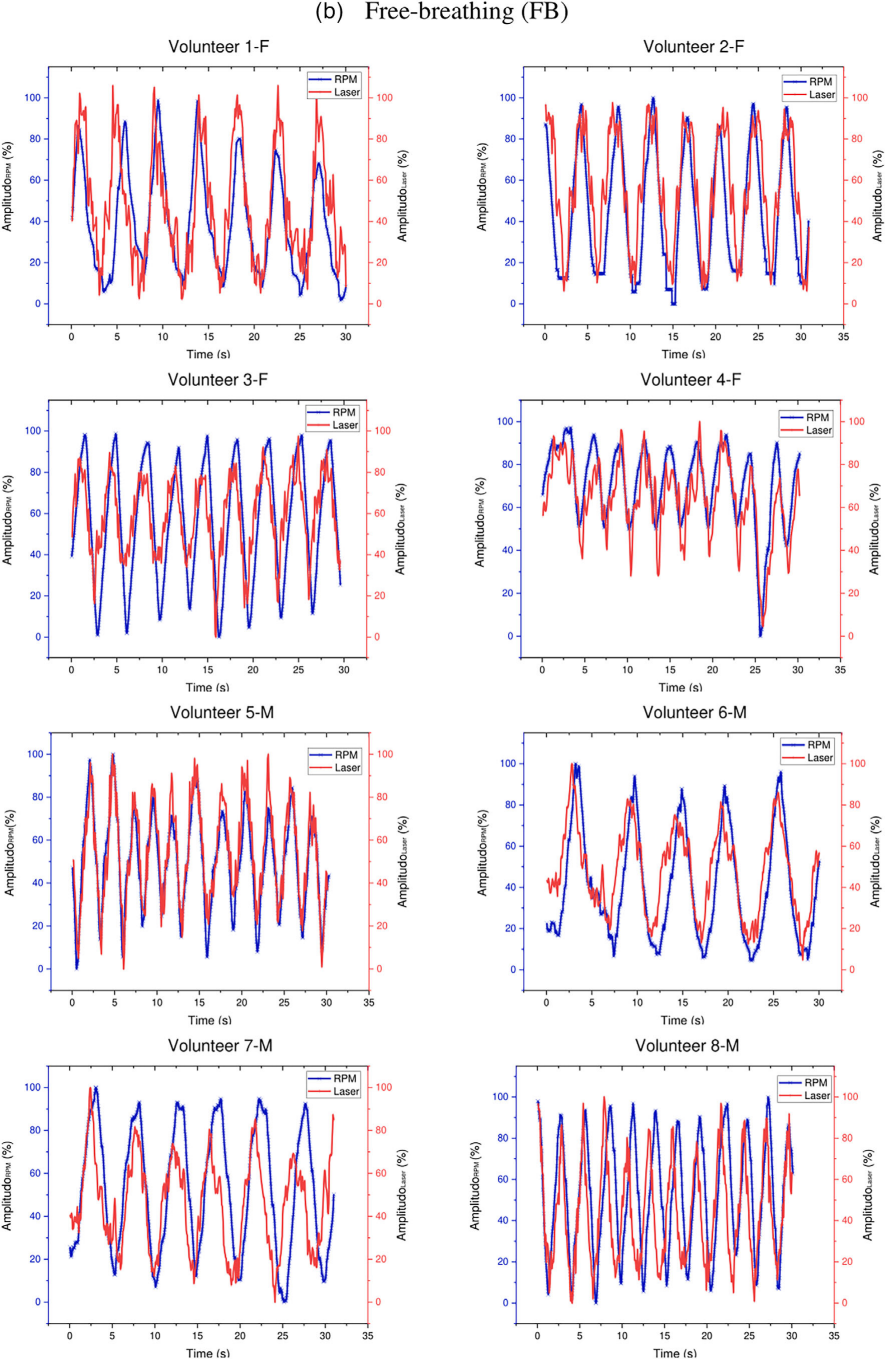

A time delay in the respiratory laser‐based system can result in an unexpected phase mismatch, as shown in Figures 9 and 12. When analyzing breath waveforms from the four volunteers collectively, a time delay of 55.3 ± 6.7 ms was observed.

## 4 DISCUSSION

### 4.1 Spatial accuracy test

This research focuses on the accuracy tests performed during the calibration stage of the laser‐based system. The spatial accuracy test conducted as a part of this study achieved an average error percentage value well below the acceptable threshold of 10%^24^ and some exceeded the manufacturer's tolerance limit of 1.5%.^25^ The test was performed, and the precision of the distance readings was validated, indicating that spatial accuracy does not represent a consistent result for various amplitudes. Consequently, manual calibration was conducted to address alignment errors in the Leuze LDS system. Calibration was performed before each measurement to derive a linear curve‐fitting equation input into the Arduino IDE. The process compensates for the laser distance displacement, thereby ensuring the accuracy and precision of the readings. Consequently, as listed in Table 4, all variations in distance data deviate by less than 1 mm, aligning with spatial accuracy standards established by Anonym^14^ and confirming the effectiveness of the laser system prototype. Based on a study by Jung‐In et al., which applied the 1 mm criterion of Acceptance Test Procedure (ATP) to evaluate the spatial integrity of the visual guidance patient‐controlled (VG‐PC) respiratory motion detection system, their approach focused on assessing how object shifted across transverse, sagittal, and coronal planes between Magnetic Resonance (MR) image and VG‐PC respiratory motion detection system.^26^ While their methodology differs from this study, it ensures that the distance reading from the laser system does not deviate from the reference system within a single plane. The result of this study clinically can be useful for accurately and consistently interpreting spatial information. This study revealed that assessing the reproducibility of the laser system involved calculating the coefficient of variation, which yielded an average of 0.3%, which did not surpass the manufacturer's tolerance limit of 0.5%.^24^ This indicates that the laser system can be considered effective due to its good precision and accuracy in detecting object movements, making it suitable as a prototype for a respiratory motion detection system. Calibration of the system is recommended, at least once a month, to minimize the possibility of reading errors that could affect the accuracy in the delineation and delivery of doses to patients in clinical.

**TABLE 4:** Deviation of distance results from the readings of LDS compared to DLS.

Distance (mm)	DLS (mm)	LDS (mm)	Deviation (mm)
2	3.99	4.05	0.06
4	8.0	8.13	0.13
6	11.98	11.77	0.21
8	15.99	15.67	0.32
10	20.0	20.17	0.17
12	23.45	23.54	0.09
14	27.49	27.16	0.33
16	31.25	30.72	0.73
18	35.42	35.62	0.2
20	39.4	39.3	0.1
22	44.08	44.50	0.48
24	48.55	48.22	0.33

Abbreviations: DLS, digital linear scale; LDS, laser distance sensor.

### 4.2 Amplitude calibration and temporal accuracy

Figure 6 illustrates the qualitative outcomes of the laser system concerning the phantom movement along the SI direction of the CIRS motion phantom. The amplitude and period curves depict the precise alignment of the laser‐tracking system with the motion of the CIRS phantom. Despite the increasing object movement, the correlation coefficient decreases quantitatively and the error increases. However, the correlation coefficient in this study remains high even with the largest measurement of 20 mm. This illustrates that the in‐house laser‐based system performed well when reading object movements. Further investigations with more variation and smaller intervals are required to substantiate this observation. Table 2 indicates the inverse relationship between the object movement magnitude, correlation coefficient, and error. The error in amplitude calibration deviates from 0.02% of Kim et al. by varying the same amplitude calibration, specifically by 0.2%.^12^ This deviation may stem from the imprecise positioning of the laser perpendicular to the CIRS phantom surface and the phantom surface color. Vukasinovic et al. emphasized the impact of the object surface color on laser reading systems, attributing different readings to varying surface colors at the same distance. Therefore, we calibrated the laser system readings by altering the color of the phantom surface. The calibration aimed to validate the amplitude calibration results, acknowledging the potential influence of the surface color and affirming the findings of Vukasinovic et al.^18^ This step is crucial, as patient skin tones vary, and it is necessary to validate assertions regarding the surface color impact.

The accuracy of the LDS readings in this study decreased with the darker surface colors of the phantom. This decline is attributed to the increased absorption of laser light by the darker surfaces, resulting in less light being reflected by the LDS receiver. However, laser light penetrates when the surface is transparent. The relationship between the reflected laser light and the optical properties of the surface is contingent on the angle of incidence of the laser light. Vukasinovic et al.^18^ showed that white surfaces exhibit optimal reflection, followed by red, green, and blue surfaces. AAPM TG 302 gave a recommendation to use light‐colored for the best monitoring results, as surface color has an impact on localization accuracy.^10^ An incident laser introduces various possibilities, including transmitted laser light, absorption, diffuse reflection, and specular reflection.^27^ This study examined color variations using three colors similar to the general skin tone. The results show good correlation coefficients and low error percentages. In cases where a patient's skin is slightly darker than the three colors tested, which are #F8F8FF, #FFFAF0, and #FDF5E6, and lighter than #000000, it is recommended to use micropore tape in the area where the laser will be applied because we used it on top first color. This helps reduce laser beam absorption and ensure accurate laser readings.

The temporal accuracy of the laser in detecting movement is crucial during clinical procedures. If the timing of the laser reading does not match the actual movement of the target, incorrect exposure may occur. Therefore, it is crucial to ensure that the timing of the periodic laser is precise to avoid any discrepancies in the breath curve readings. The temporal accuracy obtained in this study remains below the specified temporal accuracy threshold of 0.1 s, as outlined in AAPM TG 142.^19^ These guidelines assume object movement at speeds that do not exceed 20 mm/s.

Therefore, based on the spatial, amplitude, and temporal accuracy tests, it can be concluded that the LDS laser system demonstrates good performance.

### 4.3 Volunteer testing of the laser‐based system

Volunteer testing involved eight volunteers: four healthy males and four healthy females, as shown in Figures 9 and 12. Visual analysis of the results revealed that the volunteer breathing patterns during inhalation and exhalation for both DIBH and FB exhibit similar trends, whether measured in the abdomen or the inferior end of the sternum area. Although slight differences exist in amplitudes, the overall patterns remain consistent. Volunteer tests in this study were carried out with relative normalization of the volunteer amplitude from 0% (end‐expiration) to 100% (end‐inspiration)., rather than absolutely in millimeter, for both the laser‐based prototype and the clinical respiratory motion detection system as shown in Figures 7, 9, and 12. The rationale behind this choice is to simplify the visual analysis, as the clinical Anzai system reads relative distances rather than absolute values. Absolute normalization is challenging because of the inherent nature of a clinical Anzai system that operates based on relative distances. In contrast to the approaches taken by Kim et al.,^12^ who normalized the average by approximately 20 mm, and Chang et al.,^28^ who evaluated the respiratory motion detection system by normalizing the relative amplitude at 28 mm as the maximum amplitude for their patients, this study adopted a different normalization strategy.

As shown in Figure 9, male subjects with DIBH typically exhibit predominantly diaphragmatic breathing, unlike female subjects. Abdominal breathing and diaphragmatic breathing are essentially the same, as both involve the diaphragm and describe deep breathing techniques that help engage the lower lungs. Diaphragmatic breathing involves taking deep breaths that expand the lungs downward into the diaphragm, rather than relying solely on the abdomen or ribcage. Typically, diaphragmatic breathing results in a deeper expansion of the abdomen (below the ribs), while abdominal breathing involves a more superficial expansion of the abdomen, with less focus on fully engaging the diaphragm.^29^ The breathing pattern for both the chest and abdomen was significantly more stable and greater in male subjects compared to female subjects, who showed a gradual decline during DIBH. Additionally, the breathing pattern at the inferior end of the sternum was more stable in males than in females, as shown in Figure 12. Our findings align with previous studies by Kaneko et al.^30^, ^31^ Figure 12 shows that the amplitude of the RPM is higher than that of the laser because of the influence of the position. The point of interest of the laser is approximately 1–2 cm from the position of the RPM box marker, as shown in Figure 4. The position of the laser point on the inferior end of the sternum interferes with the movement of the heart. The ventricles of the heart are located between the sternum and the vertebral column.^32^ This positioning can interfere with the breath readings of laser‐based systems, potentially resulting in inaccurate breath results. Therefore, it is crucial to determine the best position for measuring the respiratory motion as a surrogate target.

The breath measurement location and distinct clinical respiratory motion detection systems influenced these correlation coefficient variations as shown in Figures 10a and 11a. The RPM system, which employs an infrared system to detect the marker box movement in the chest, differs from the Anzai system, which utilizes a pressure system integrated into the abdominal belt. The results suggest that the laser‐based system performs optimally in the abdomen compared with the chest for free‐breathing and DIBH. The DIBH and FB patterns remained stable in the abdominal area, and the correlation coefficients were not significantly different. This finding aligns with those of Giraud and Houle,^33^ where it was observed that the largest breath motion occurred between the xiphoid and umbilicus in the abdomen, a region often recommended in RPM system protocols.^34^ According to this study, the average correlation coefficient of free‐breathing on chest measurements in female volunteers is slightly higher than that in male volunteers. In contrast, the average correlation coefficient for the abdomen of male volunteers is significantly higher than that of female volunteers. Chu et al.^35^ conducted a study comparing a spirometer with a strain sensor and found that the chest measurements of female volunteers had a higher correlation coefficient than those of male volunteers. Meanwhile, the abdominal measurements of male volunteers had a correlation coefficient of more than 0.9 but was less than that of female volunteers. Figures 10b and 11b show that the RMSE error values remain below 1 mm for FB and DIBH measurements in both the abdomen and the chest.^26^ This indicates that the discrepancies or differences in readings between the prototype of the laser‐based system and the RPM and the Anzai systems are still within the tolerance limit. Therefore, the performance of the prototype of laser‐based system can be considered quite effective in detecting respiratory motion, similar to existing clinical respiratory motion detection systems.

The time delay for the respiratory motion detection system is determined by comparing the motion curves and position of a moving object with the beam on‐off time.^36^ The assessment of the time delay was aimed at understanding the responsiveness of the respiratory motion detection system to target surrogate movements, particularly in the chest and abdomen. This delay can lead to treatment inefficiencies and misses in target regions. When the beam is on and the laser system is at a certain amplitude limit, the period when entering and leaving the limit must match the period of the target motion. The expectation is for the amplitude to remain stable both during simulation and throughout the treatment process. Cone Beam Computerized Tomography (CBCT) 4D (symmetry) can be utilized in clinical settings to confirm this consistency. Further research is necessary to ensure that the movement of the target concerning a fixed reference point remains accurate during both the simulation and treatment phases with the integration of this prototype system with CT and LINAC systems.

In this study, the clinical respiratory motion detection system response was considered a reference and assumed to align with the target surrogate's motion. Time‐delay analysis was performed using a Python application with a fast Fourier transform function code. The time delay in this study remained within acceptable limits, being below 90 ms. It is crucial to note that Keall et al. defined a time delay of 90 ms as the delay between the fiducial marker and the onset of irradiation using fluoroscopy.^2^ According to the AAPM TG 142 report, the time delay should not exceed 100 ms for a tumor movement speed of 2 cm/s. Therefore, measuring the time delay before implementing a laser‐based system in radiotherapy of the respiratory motion detection system is crucial. However, the laser‐based system is still in its early stages; therefore, the observed time delay may not fully represent the delay throughout the clinical treatment process.

This study only measured motion in the anterior‐posterior (AP) direction, which means that it did not fully represent the actual target movement. A previous study by Keall et al.,^2^ observed breathing motions in 3D, including SI, AP, and lateral motions. To address this limitation, further research, which involves measuring motion in the SI, AP, and lateral directions using a camera. The camera is used to measure the SI and lateral movements, enabling the prototype laser‐based respiratory motion detection system in this study to read the respiratory motion with three degrees of freedom.

## 5 CONCLUSION

The laser‐based respiratory motion detection system demonstrated effective performance and clinical acceptability based on calibration data. This study exhibited a commendable correlation with existing clinical respiratory motion detection systems based on volunteer testing results. The ability of the system to measure and utilize a limited amount of volunteer data in both the chest and abdominal areas suggests that it has potential utility in delivering radiation treatment to thoracic and abdominal cancer areas affected by respiratory movement, with a high correlation coefficient and lower error percentage. However, laser‐based respiratory motion detection systems are still nascent and currently only measure movement in the AP direction. The color of the surface object can affect the results of laser reads. However, the sample size of volunteers in this study was relatively small; therefore, additional studies on the number of subjects should be conducted. Intensive research is essential to establish the integration of this laser‐based respiratory motion detection system with the linear accelerator (linac) and computed tomography (CT) scan systems to ensure seamless and comprehensive application in clinical settings. In addition, direct observation of the tumor motion during treatment is required to minimize uncertainties in the displacement and phase relationship between the chest or abdominal surrogate and the tumor.

## AUTHOR CONTRIBUTIONS

Isnaini Nur Islami was responsible for data collection, data analysis, and manuscript writing. Amar Ma'ruf Irfan Muhamadi contributed to data collection, device design, data analysis, and manuscript writing. Wahyu Edy Wibowo contributed to data collection. Aloysius Mario Yudi Putranto contributed to data collection. Arief Sudarmaji contributed to research design and device design. Fielda Djuita contributed to data collection. Supriyanto A. Pawiro was responsible for research design, data collection, data analysis, and manuscript writing.

## CONFLICT OF INTEREST STATEMENT

The authors declare no conflicts of interest.

## ETHICS STATEMENT

This study included healthy human subjects and was approved by the Ethics Committee of the Faculty of Medicine Universitas Indonesia (KET‐1292/UN2. F1/ETIK/PPM.00.02/2022).
